# Utterance Level Feature Aggregation with Deep Metric Learning for Speech Emotion Recognition

**DOI:** 10.3390/s21124233

**Published:** 2021-06-20

**Authors:** Bogdan Mocanu, Ruxandra Tapu, Titus Zaharia

**Affiliations:** 1 Department of Telecommunications, Faculty of ETTI, University “Politehnica” of Bucharest, 060042 Bucharest, Romania; bcmocanu@comm.pub.ro; 2 Institut Polytechnique de Paris, Télécom SudParis, Advanced Research and TEchniques for Multidimensional Imaging Systems Department, 9 rue Charles Fourier, 91000 Évry, France; titus.zaharia@telecom-sudparis.eu

**Keywords:** deep convolution neural networks, speech emotion recognition, emotion metric learning, utterance level feature aggregation

## Abstract

Emotion is a form of high-level paralinguistic information that is intrinsically conveyed by human speech. Automatic speech emotion recognition is an essential challenge for various applications; including mental disease diagnosis; audio surveillance; human behavior understanding; e-learning and human–machine/robot interaction. In this paper, we introduce a novel speech emotion recognition method, based on the Squeeze and Excitation ResNet (SE-ResNet) model and fed with spectrogram inputs. In order to overcome the limitations of the state-of-the-art techniques, which fail in providing a robust feature representation at the utterance level, the CNN architecture is extended with a trainable discriminative GhostVLAD clustering layer that aggregates the audio features into compact, single-utterance vector representation. In addition, an end-to-end neural embedding approach is introduced, based on an emotionally constrained triplet loss function. The loss function integrates the relations between the various emotional patterns and thus improves the latent space data representation. The proposed methodology achieves 83.35% and 64.92% global accuracy rates on the RAVDESS and CREMA-D publicly available datasets, respectively. When compared with the results provided by human observers, the gains in global accuracy scores are superior to 24%. Finally, the objective comparative evaluation with state-of-the-art techniques demonstrates accuracy gains of more than 3%.

## 1. Introduction

Speech action is a rich and dense form of human communication that includes both linguistic and paralinguistic elements of information. The former refers to the verbal content itself, while the latter concerns the manner in which the speech action is performed and involves aspects related to facial expression, gesture, intonation and pitch. All these elements contribute to the emergence of the concept of emotion that is a characterization of the affective, psychological state associated with a given speech action. Emotion plays an import role in identifying the intention, the empathy and, in some cases, even the meaning of the spoken message. 

In this paper, we specifically tackle the issue of emotion identification exclusively from audio features. Emotion characterization first requires the consideration of a generic emotional model. The extensive research carried out in the last few decades in various fields, including psychology, neurosciences and medicine, makes clear various emotional models—both continuous and discrete [1]. 

Among the most popular continuous emotion representations let us mention the 2D activation–valence model [2]. Activation (or arousal) quantifies the amount of energy required to express a certain emotion. For example, anger or joy involves a high energy pitch, while sadness is rather characterized by low energy values. On the contrary, valence describes the degree of positivity/negativity of an emotion. For example, the valence can be used to distinguish between emotions such as being angry or happy, where activation is high in both cases. 

In the case of discrete emotion representations, the emotions are quantified into a set of discrete categories. In their pioneering work [3], Eckman and Friesen identified a set of six basic, universal emotions (i.e., anger, disgust, fear, happiness, sadness and surprise) that humans perceive in a similar manner, regardless of their regional, ethnical or cultural differences. Contempt was subsequently added as one of the basic emotions [4]. Since then, numerous other models have emerged, but this issue is out of the scope of our work. 

The automatic recognition and identification of emotions from speech is a task that is naturally performed by humans in everyday life. However, it is highly challenging to design fully automatic speech emotion recognition (SER) approaches that are able to detect complex emotions from natural speech. The information included in the audio signal depends on multiple factors, including sample duration, speaker accent, gender or language. In addition, the emotion recognition process is highly subjective because people can interpret emotions differently depending on various factors, such as culture, environment, or state of mind [5]. Moreover, labeling speech utterances with the suitable emotion during data annotation is also subject to human perception.

An important issue to be considered concerns the temporal level of granularity at which the emotion should be recognized. Emotion is not an instantaneous characteristic that can arbitrarily fluctuate from one instant to another, but rather a stable affective state over a whole sentence (or set of sentences) expressed in a given time interval. This leads to the concept of utterance, which can be defined as a continuous piece of speech that is preceded by silence and followed by silence or a change of speaker. In the context of emotion recognition, the goal is to condense speech information into a single utterance-level representation that is able to capture the speech dynamics. This feature aggregation process becomes extremely important for audio signals obtained with background noise or in unconstrained conditions, where the irrelevant parts must be filtered out and their contribution to the final descriptor should be minimized.

In this paper, we notably propose a novel speech emotion recognition methodology based on GhostVLAD [6] utterance-level feature aggregation and end-to-end emotional metric learning. The main contributions of the paper are the following:

1. A deep learning-based, end-to-end speech emotion recognition technique. The method exploits the recently introduced Squeeze and Excitation ResNet architecture [7]. The challenge is to derive a fixed-length, discriminative representation at the utterance level for audio segments of arbitrary length.
2. The introduction within the aggregation process of a trainable GhostVLAD [6] layer. In contrast to [8], where a NetVLAD [9] approach is used for feature aggregation, in our case we privilege the use of ghost clusters. We show that they are able to capture noisy segments and thus obtain a more robust feature representation.
3. A learnable emotional metric which enriches the traditional triplet loss function with an additional emotional constraint. By taking into account the relations between the considered emotional categories, as defined in the Mikel’s wheel representation [10], the method makes it possible to obtain more discriminative embeddings, with more compact emotional clusters and increased inter-class separability.

The rest of the paper is organized as follows. Section 2 reviews the speech emotion recognition state-of-the-art techniques. Section 3 describes the proposed architecture and details the key elements involved. Section 4 and Section 5, respectively, present the experimental setup and the evaluation results obtained. Finally, Section 6 concludes the paper and opens some perspectives of future work.

## 2. Related Work

The performance of an SER system greatly relies on the considered features/representations and those extracted from the audio signal. Traditional model-based approaches encode the emotional speech into one global feature vector, which most often represents the statistics (e.g., mean, median, standard deviation) of some low-level, hand-crafted audio primitives. Such representation is defined either in the temporal, spectral or time-frequency domains [11]. The audio descriptors involve features that are related to the speaking rate [12,13,14], spectral features, such as such as coefficients (MFCCs), linear prediction cepstral coefficients (LPCCs), modulation spectral features [15,16,17] or voice quality features (e.g., shimmer, jitter or normalized amplitude quotient) [18,19,20]. A detailed comparison between various hand-crafted audio features is reported in [21]. The main limitation of such global-level acoustic representations is related to their limited capability of capturing the speech variation dynamics along a given utterance segment [22].

Notably, the rest of this chapter focuses on emotion recognition methods, based on pattern recognition algorithms that are able to automatically learn relevant emotional features from a given training dataset. The majority of the reported state-of-the-art works aim at identifying a discrete set of emotional categories, more or less inspired from Eckman’s taxonomy (sub-sets or super-sets).

Within this context, the feature representation is used to train different machine learning systems, such as support vector machines [23,24,25], 1D-convolutional neural networks (1D-CNN) [26,27], 2D-CNN [28,29,30,31,32] or recurrent neural networks (RNN) [33,34,35,36].

In [24], joint spectro-temporal features are extracted from the audio signal and are applied to detect the emotional status of noisy speech segments using a linear kernel SVM classifier. Similarly, in [23], a multi-modal emotion recognition system based on deep SVM techniques is introduced. The framework uses both audio-visual sources of information for emotion recognition purposes. The novelty of the approach relays on the SVM classifier extended with deep metric learning that jointly obtains a discriminative score and a robust representation in the latent space of both modalities. In [25], the authors propose an analysis of the speech data using machine learning techniques in order to extract low-level features such as mel-frequency cepstral coefficients, delta and delta–delta MFCCs and spectral centroids. The reported experimental results show that ensemble learning yields superior performances when compared to single estimators. However, the SVM classifier does not perform well when the data set is noisy (i.e., target classes are overlapping) or when the emotion datasets are large.

With the recent advances in deep learning techniques, the scientific community has focused its attention on developing SER systems that can identify emotions without any human intervention. Such techniques, also called end-to-end systems, prove to be much more independent, regarding the choice of features, than the model-based approaches. A 1D-CNN framework that takes the mel-frequency cepstral coefficients, chroma-gram, mel-scale spectrogram, Tonnetz representations and spectral contrast features as inputs is proposed in [26]. The approach works directly with raw audio data without the need for any visual information. Another 1D-CNN approach is proposed in [27]. The system is used to detect events and classify emotions over long range audio–visual datasets. As a general observation, it can be highlighted that the CNN systems are intensively used for speech emotion recognition due to their ability to learn low level feature descriptors from raw data [37].

Another family of approaches concerns 2D-CNN techniques. In [28], the authors propose inferring salient features by using 3D convolutional neural networks (2D-CNN). The CNN training process is performed in two successive stages. Initially, the unlabeled samples are used to learn local invariant features (LIF) using a sparse auto-encoder with reconstruction penalization. Secondly, the LIF are used as inputs to a feature extractor using a novel objective function that encourages feature saliency. The method can disentangle affective salient features from other noisy factors, such as speakers and language. In addition, the system shows superior performance when compared with several state-of-the-art hand-crafted feature representations. Ma et al. [29] propose an emotion-pair framework that constructs discriminative feature subspaces for every two different types of emotions (emotion-pair) in order to generate more precise emotion bi-classification results. The authors observe that, in the high dimensional emotion spaces, the distances between some archetypal emotions are closer than the others (e.g., happy and angry are closer than happy and sad, indicating that happy is more similar to angry than to sad). Based on this observation, a 2D-CNN classifier with decision fusion is proposed. In [30], Lian et al. propose a speech emotion recognition framework based on a Siamese network. The 2D-CNN architecture exploits a contrastive loss function in order to reduce the ambiguity of various emotions by encouraging intra-class compactness and inter-class separability. The experimental evaluation is performed upon multiple features and pairwise selection mechanisms. The gains in accuracy scores obtained when compared with traditional Siamese network with cross entropy loss are of 2.55%. 

In order to take advantage of the high robustness of deep convolutional neural networks, in [31], a pre-trained 2D-CNN is used in the context of speech emotion recognition. The CNN is designed to extract low-level features from state-of-the-art speech emotional datasets. Subsequently, a correlation-based selection technique is applied in order to determine the most appropriate and discriminative features that can be exploited for SER purposes. Concerning the emotion classification itself, various machine learning algorithms are evaluated, including SVM, random forests, k-nearest neighbor and neural network classifiers. In [32], end-to-end neural network embedding, based on triplet loss and residual learning, is proposed. Embedding is learnt from the emotional information of speech utterances and is based on the ResNet (Residual Neural Networks) [38] architecture. The 2D-CNN is trained using soft-max and triplet-loss functions. The similarity between various types of emotions is determined by computing the cosine distance between low-level feature representations. Recently, an utterance-level representation for SER has been introduced [8]. A NetVLAD layer is used here as a trainable discriminative clustering method in order to aggregate frame-level descriptors into a compact, single-utterance-level vector. In addition, in order to reduce the influence of unbalanced emotional classes, unigram label smoothing with prior emotional class distribution is proposed. Aside from their effective means of identifying emotions, the methods based on CNN present some limitations. The CNNs are able to learn low-level feature representation from high dimensional data but are highly sensitive to small variations and distortions. In addition, the corresponding CNN models require large memory capabilities.

In [33], a deep learning method for automatic identification of emotionally relevant features from speech is proposed. The considered RNN (Recurrent Neural Network) learns a set of short-time, frame-level acoustic features that are emotionally relevant. In addition, the salient features are obtained using a novel strategy of feature pooling over time. A multi-modal emotion recognition approach from video streams is introduced in [34]. A long–short time memory recurrent neural network (LSTM-RNN) is used here in order to incorporate knowledge about emotion evolution over time and to derive various cues from isolated pitch signals. The low-level feature descriptors extracted from both the audio and video streams are concatenated and fed to a support vector machine (SVM) classifier in order to obtain the final prediction. Another SER framework, based on RNN, that incorporates both low-level descriptors (local features) and statistical characteristics, (global features) is introduced in [35]. The extracted features are used to determine emotions at various temporal scales, including frame, phoneme, word and utterance. The experimental evaluation reported shows that speech utterances varying from 2 to 5 secs return the highest accuracy scores. A triplet loss framework, based on LSTM, is presented in [36]. The system learns mapping from the acoustic to discriminative embedding features, which are further used to train an SVM. The model jointly and simultaneously uses triplet loss and supervised loss. Triplet loss is designed to reduce the intra-class distances, while increasing the inter-class distances. The supervised loss incorporates the class label information into the decision. In order to deal with variable speech inputs, three different strategies are evaluated (cycle, repeat and pad mode). 

In a general manner, the state-of-the-art analysis highlights that the existing emotion recognition approaches still leave room for improvement with respect to their accuracy scores and heavily depend on the quality of the training datasets. Most emotion datasets consist of elicited or acted speech segments, typically created in a studio, where the actors read a written text. Another difficulty in emotion speech analysis is that the utterances have variable length. Some approaches obtain equal length inputs by clipping the utterance, meaning that longer speech segments are cut while the shorter ones are zero-padded. Most authors propose obtaining a feature representation of fixed dimension for every sample, based on various aggregation techniques. 

The emotion recognition methodology, proposed in this paper, notably aims at overcoming these limitations and difficulties. The following section describes the proposed approach and details the various modules involved. 

## 3. Proposed Approach 

**Figure 1:**
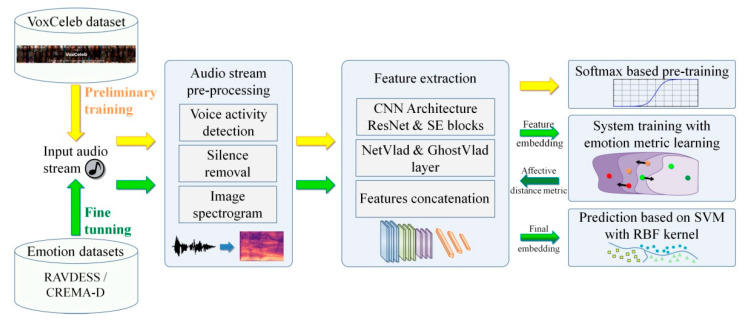
The proposed methodological framework with the main steps involved: audio-stream preprocessing, feature extraction and utterance level aggregation, system training with emotion metric learning and SVM training.

Figure 1 presents an overview of the proposed architecture, which involves three different parts: audio pre-processing, audio feature extraction and the emotion metric learning.

### 3.1. Audio Signal Pre-Processing

**Figure 2:**
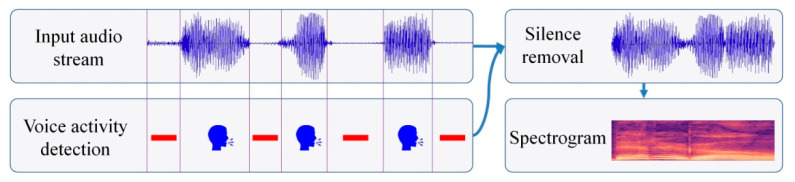
Audio signal pre-processing: voice activity detection, silence removal and spectrogram image computation.

A pre-processing step is initially applied to each audio segment, which consists of voice activity detection and silence removal [39]. The clean segments are regrouped into a single audio stream, represented with the help of a spectrogram image (Figure 2). The spectrogram is generated using a sliding Hamming window of width 32 ms and an offset step of 10 ms. We have used the FFT implementation proposed in [40] with 512 input samples, which yields 256 frequency components that, together with the DC component, form the 257 frequency components of the resulting STFT (Short-Time Fourier Transform). Finally, the spectrograms are zero-meaned and normalized by their standard deviation.

Thus, the obtained spectrogram is a (257×T) matrix, with T being the number of time instants of the considered audio segment. Such an image-like representation is naturally adapted for further processing with a convolutional neural network. However, the output of this feature extractor is a variable-length feature vector, which is dependent on the temporal length of the input utterance. 

In a first stage, in order to boost the discriminative power of the representation, we have adopted a modified version of the Squeeze and Excitation ResNet-34 architecture [7] that is here modified in a fully convolutional way in order to encode 2D spectrograms. Compared to the standard ResNet, we cut down the number of channels in the residual block. The CNN architecture maps the input spectrogram of size 257×T×1 onto a set of T/32 feature descriptors of size 512. The dimension reduction from T to T/32 is given by two max-pooling operations and three convolution operations, with stride 2 applied at the beginning of each convolutional block, while 512 represents the number of filters of the last convolution layer. This approach makes it possible to derive, starting from a global representation, a semi-local one, which is subsequently adaptively learnt. At the end of this process, the audio signal is described by a number of T/32 feature descriptors. Each segment contributes, in a different manner, to the global feature descriptor by taking into account the temporal dynamics of the audio signal.

This representation still has a variable length, which depends on the duration T of the considered audio segment. However, it is intrinsically well-adapted for deriving a globalized, fixed-length representation. In our work, we have retained a GhostVLAD [6] feature aggregation layer, which is described in the following section. 

### 3.2. GhostVLAD Feature Aggregation 

**Figure 3:**
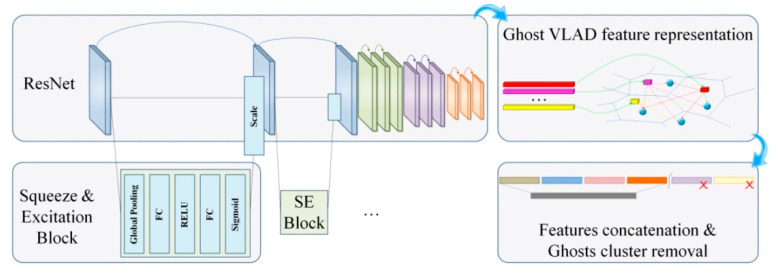
SE-ResNet CNN extension with a GhostVLAD layer for feature aggregation.

GhostVLAD is a recent extension of the NetVLAD technique previously introduced in [9]. In its original form, NetVLAD can be interpreted as a trainable discriminative clustering technique. Here, every segment-level descriptor is softly assigned to different clusters, and their residuals are encoded as output features. The NetVLAD layer receives, as an input, M=T/32 dense descriptors ${\left\{p_{i}\right\}}_{i=0}^{M-1}$, taken from the last convolutional layer of the considered SE-Resnet model and yields a single (K×D) dimensional matrix of speech descriptors **V**, where D represents the size of each input descriptor $p_{i}$ (in our case, *D* = 512), and *K* is the number of considered clusters. More precisely, the NetVLAD descriptor matrix ***V*** stores the cumulated differences (residuals) between the feature descriptors and the clusters’ centroids. The GhostVLAD technique further extends the NetVLAD, with the introduction of a number of *G* dummy clusters, so-called ghosts. The ghost clusters contribute to the soft assignment of the individual descriptors as any regular ones (Figure 3). However, their corresponding residuals are discarded from the final output descriptor. The matrix **V** can be computed as follows:

(1) $$V\left(j,k\right)=\overset{M}{\underset{i=1}{\sum }}\frac{e^{w_{k}^{T}\cdot p_{i}+b_{k}}}{\sum_{l=1}^{K+G}e^{w_{l}^{T}\cdot p_{i}+b_{l}}}\left(p_{i}\left(j\right)-c_{k}\left(j\right)\right)$$

where $p_{i}\left(j\right)$ and $c_{k}\left(j\right),\;\mathrm{respectively},$ denote the *j*-th component of the *i*-th descriptor and the *k*-th cluster center, $\left\{w_{k}\right\}$ and $\left\{b_{k}\right\}$ are trainable parameters, and k∈[1, 2, …, K]. The term $w_{k}$ represents the soft assignment of the individual components of the input vector $p_{i}$ to the k cluster, while $b_{k}$ is a bias term. Each *D*-dimensional column of the **V** matrix stores the sum of residual $\left(p_{i}-c_{k}\right)$ of descriptors assigned to cluster $c_{k}$. The matrix V(j,k) is first L_2_-normalized column-wise, and, subsequently, L_2_-normalized entirely. 

The incorporation of ghost clusters allows the network to adjust the contribution of each example to the template representation by assigning examples to be ignored to the ghost clusters. In this way, it becomes possible to increase the robustness of the recognition process, by discarding irrelevant samples.

In the ideal case, the role of the ghost clusters is to capture the noisy speech inputs, making their contribution to non-ghost clusters close to zero, and thus annihilate their effect to the final descriptor representation. We need to highlight that we do not explicitly force noisy/confusing speech segments to assign to ghost clusters, but instead we let the network discover the optimal behavior through end-to-end training for emotion recognition. We thus obtain a *D* × *K* fixed-length descriptor that globally describes the analyzed speech segment at the utterance level.

This entire process can be interpreted as an embedding method that encodes a given audio segment *x* into a feature space **V** = *f*(*x*)$\;\in {\mathbb{R}}^{D\times K}$. Of course, the learned embedding can be directly used for emotion classification purpose by considering, for example, a softmax output layer.

In our work we have considered a different emotion-constrained optimization function that takes into account in a finer manner the relations between emotions. 

The principle consists of designing a loss function that forces the embedded features **V** = *f*(*x*) to fulfill a set of constraints in terms of relative distances. The objective is to ensure that the feature extractors yield similar representations for the embeddings assigned to the same (or similar) emotion categories and distant representations for samples belonging to different classes. In terms of methodology, this requires the specification of an adapted loss function to be used when training both the SE-ResNet-34 and GhostVLAD layers.

The proposed emotion metric extends the triplet loss function with an emotional constraint, as described in the following section.

### 3.3. Emotion Metric Learning

**Figure 4:**
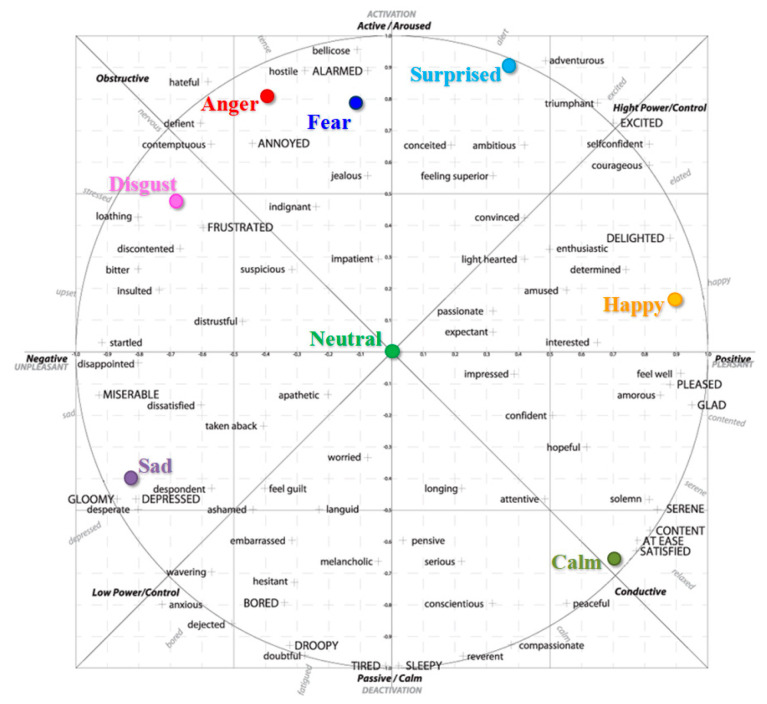
Emotion representation in the valence-arousal space using Mikel’s wheel of emotions.

In order to define the relationships between emotions, we considered the 2D valence-arousal emotional model, described by the Mikel’s wheel of emotions (Figure 4). With respect to the Eckman and Friesen discrete set of emotions, we can identify four negative (i.e., sadness, fear, disgust and anger) and three positive emotions (i.e., surprise, happiness and calmness). As it can be observed, the neutral state is situated in the center of the wheel with zero valence and arousal scores.

We propose integrating, within the triplet loss function, a component describing the sentiment constraint by taking into consideration the relations between various emotions. In its traditional formulation, the triplet-loss constraint usually generates mini batches of triplets ($x_{i}^{a},\;x_{i}^{p},\;x_{i}^{n}$ ), with: $x_{i}^{a}—$anchor, $x_{i}^{p}$—positive instance (sample belonging to the same class) and $x_{i}^{n}$—negative instance (sample belonging to a different class), with $(x_{i}^{a},\;\;x_{i}^{p}$,$\;x_{i}^{n})$ ∈ Γ, where Γ is the set of all possible (anchor, positive, negative) triplets in the training dataset. 

The goal is to learn a metric that assigns a smaller distance to images belonging to the same class, while maximizing the distance to other categories, as described in the following equation:

(2) $$D\left(x_{i}^{a}\;,\;x_{i}^{p}\right)+\alpha <D\left(x_{i}^{a}\;,\;x_{i}^{n}\right)$$

with:

(3) $$D\left(x_{i},x_{j}\right)=||f\left(x_{i}\right)-f\left(x_{j}\right){\left|\right|}_{2}^{2}$$

where $f\left(x_{i}\right)$ and $f\left(x_{j}\right)$ are the embedding feature representations associated with samples $x_{i}$, and $x_{j}$, respectively, and α>0 is a margin parameter that enforces the separation between positive and negative pairs. 

Then, the triplet loss that needs to be minimized can be expressed as follows:

(4) $$Loss=\overset{N}{\underset{i=1}{\sum }}{\left[D\left(x_{i}^{a}\;,\;x_{i}^{p}\right)-D\left(x_{i}^{a}\;,\;x_{i}^{n}\right)+\alpha \right]}_{+}$$

where ${\left[m\right]}_{+}=\max\left(m,\;0\right)$ and N is the cardinality of the training dataset.

Within this context, we propose extending the traditional triplet loss with an emotion constraint. For this purpose, we introduce an additional element of so-called related emotions. With respect to the anchor, the related emotions are defined as negative samples that have the same polarity as the anchor. The objective, then, is to force the loss function to yield distances between the anchor and the relative samples that are higher than those between the anchor and the positive samples but are inferior to those between the anchor and the purely negative (i.e., different from the anchor categories and with opposite polarity) samples.

**Figure 5:**
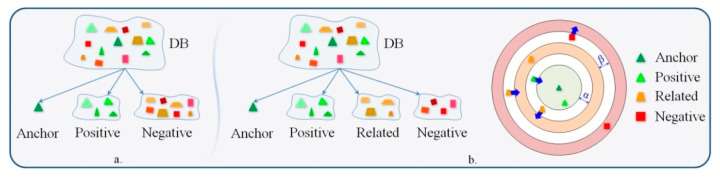
Difference between the triplet loss function and the emotion constraint. (**a**) The triplet loss function, and (**b**). The proposed emotion metric.

This principle is illustrated in Figure 5. In contrast to the existent triplet constraint that involves anchors, positive and negative samples (Figure 5a), the proposed emotion constraint consists of anchors—positive, related and negative samples (Figure 5b)—and is, thus, able to take into account the natural polarities of emotion labels. 

Mathematically, the emotion constraint (Figure 5b) can be formulated as described in Equations (5) and (6):

(5) $$D\left(x_{i}^{a}\;,\;x_{i}^{p}\right)+\alpha <D\left(x_{i}^{a}\;,\;x_{i}^{r_{1}}\right)$$

(6) $$D\left(x_{i}^{a}\;,\;x_{i}^{r_{2}}\right)+\beta <D\left(x_{i}^{a}\;,\;x_{i}^{n}\right)$$

where α>0 and β>0 are the control margins between various positive, related and negative samples.

Finally, the emotion loss function is defined as:

(7) $$Loss_{emotion}=\overset{N}{\underset{i=1}{\sum }}{\left[D\left(x_{i}^{a}\;,\;x_{i}^{p}\right)-D\left(x_{i}^{a}\;,\;x_{i}^{r_{1}}\right)+\alpha \right]}_{+}+\overset{N}{\underset{i=1}{\sum }}{\left[D\left(x_{i}^{a}\;,\;x_{i}^{r_{2}}\right)-D\left(x_{i}^{a}\;,\;x_{i}^{n}\right)+\beta \right]}_{+}$$

In the case of a neutral emotion, no related examples were identified. Therefore, for this particular class, the traditional triplet loss function is utilized. 

Regarding the training sample selection, even for small emotion datasets the number of pairs is overwhelming. Therefore, it is crucial to select a hard set of samples that violate the emotion constraints defined in Eq. 5 and 6. In our implementation, we decided to select the elements that violate the hard constrains within mini batches of features.

**Figure 6:**
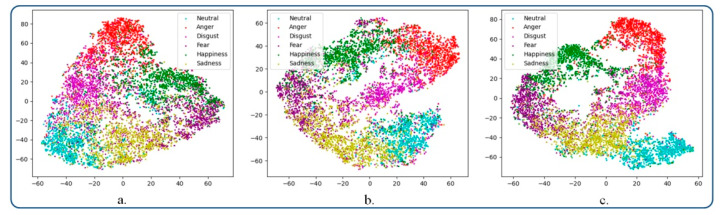
Visualization of the feature embedding using t-SNE on the CREMA-D dataset: (**a**) softmax loss, (**b**) triplet loss, and (**c**) emotion metric learning.

Figure 6 shows the sample features distribution on the CREMA-D dataset. Solely for visualization purposes, a 2D representation obtained with t-SNE embedding was used. Figure 6a presents the sample feature distribution when applying the softmax loss function. We can observe that the emotional classes are highly correlated and hence form similar clusters. 

**Figure 7:**
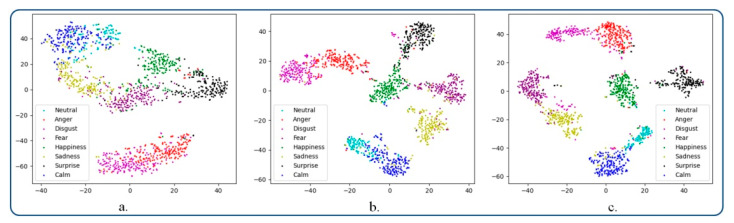
Visualization of the feature embedding using t-SNE on the RAVDESS dataset: (**a**) softmax loss, (**b**) triplet loss, and (**c**) emotion metric learning.

In Figure 6b we illustrate the feature space after applying the triplet loss function. It can be highlighted that the triplet loss improves the cluster forming, leading to better separated emotions in the novel sub-space. However, the number of formed clusters is not well defined. Figure 6c depicts the sample distribution within the feature space when using the proposed emotional constraint loss. As can be observed, the emotion classes are separated in this case more effectively. In addition, the obtained emotional clusters are more compact, while the inter-class variance is increased. Finally, in order to determine the degree of generalization of the proposed deep metric loss function, we conducted a similar experiment as above, this time on the RAVDESS dataset. A similar behavior can be observed (Figure 7). Let us note that the clusters are better structured and that the emotion classes can be clearly identified.

Following metric computation, we applied the obtained feature vectors as inputs to an SVM classifier with an RBF kernel. Let us note that other classification approaches are also possible at this stage (e.g., inclusion of a softmax layer on top of the network at the test stage), but this is not a critical choice. 

## 4. Experimental setup

In order to demonstrate the efficiency of the proposed method, we conducted an extensive experimental evaluation, carried out on two emotion-recognition datasets: RAVDESS [41] and CREMA-D [42].

### 4.1. Dataset Selection

The RAVDESS dataset [41] is a gender-balanced database consisting of 24 professional actors, vocalizing lexically matched statements in English. The dataset contains 1440 samples with eight basic emotions: calm, happy, sad, angry, fearful, surprised, neutral and disgusted. Each expression is produced at two levels of intensity (strong and normal), except for the neutral one, which is performed only at the normal level. All media formats are available: face-and-voice, face-only and voice-only. In our experiments, we considered exclusively the voice-only elements.

The CREMA-D dataset [42] consists of facial and vocal emotional expressions in sentences with six basic emotion labels: happy, sad, anger, fear, disgust and neutral. The database is composed of 7442 clips spoken by 91 actors (43 females and 48 males). Their ages ranged between 20 and 74 with diverse ethnic backgrounds (African American, Asian, Caucasian and Hispanic). The actors were asked to speak 12 utterances at four different levels of intensity (low, medium, high and unspecified). The human recognition of the intended emotions for the audio-only stream was 40.9%. The results were obtained on a panel of 2443 users with ages ranging between 18 and 89 years and with 40.5% males and 59.5% females. 

We used the RAVDESS and CREMA-D datasets for both the training and evaluation of the proposed emotion recognition framework, strictly focusing our attention on the audio signal. To the very best of our knowledge, there is no technical evaluation baseline for the considered datasets. In order to perform a fair evaluation, we divided the data into training, validation and testing sets, with all the samples from each speaker only belonging to particular set. We adopted a 10-fold cross validation with a split driven by the actors that are expressing the utterances. Thus, there is no overlap between the subjects’ clips: they are in either the test, validation or train datasets. The targeted percentages between the train, validation and test datasets are of 80%, 10% and 10%, respectively. The cross-validation technique is a well-known and widely used tool for evaluating the performances of a given model [43]. This makes it possible to obtain statistically consistent performance estimations and to assess how the results will generalize into an independent dataset. In order to ensure the full reproducibility of our results, the considered folds are reported in Table 1 (the native numberings of segments that are available for each database are here considered).

The performance measures reported in Section 5 are averaged on the considered 10-fold cross-validation scheme. 

### 4.2. Hardware Configuration

Model training and testing were performed on Nvidia 1080Ti GPU with 3584 CUDA cores, 88 ROPs and 11 GB of GDDR5X Virtual RAM, with Intel Core -i7-8700K CPU @ 3.70 GHz, 16 GB RAM CPU system with Ubuntu 18.04. 

### 4.3. CNN Training Details

For CNN training, we used a batch size of 32, the Adam optimizer and an initial learning rate of 0.001, which is divided by 10 at each of 20 epochs until convergence.

The direct training of deep networks on a relatively small audio emotion datasets is prone to overfitting. In order to mitigate this problem, we performed initial preliminary training on an additional dataset in order to obtain models with high capacity, thereby enhancing the performance of the emotion recognition system. To select the appropriate auxiliary data, a large-scale speaker identification dataset is used. This process makes it possible to obtain weight initialization that already captures some salient audio features.

In our work, we considered the VoxCeleb2 [44] dataset (a database with ≈ 1.1 million utterances). In the second phase, the model was fine-tuned to each of the considered emotion datasets that were retained (*conform.* Section 4.1). We observed that pre-training the model on larger speech datasets, and further fine-tuning the model on additional emotion datasets, has a positive impact on emotion recognition accuracy (*conform*. Section 5).

## 5. Experimental Results

In order to objectively evaluate the performances of the proposed approach, we considered the traditional accuracy score [45], defined as the percentage of correctly recognized utterances over the whole set of samples in the considered test set. In order to investigate the impact of each component on the emotion recognition performances, we considered the following five testing strategies:

- (S1) An emotion recognition approach that trains from scratch (i.e., with random weight initialization) the SE-ResNet architecture [7] on the considered emotion datasets. This approach will be considered as a baseline.
- (S2) An emotion recognition method that performs multi-stage CNN training (denoted by SE-ResNet with multi-stage training). In this case, the network is pre-trained on the VoxCeleb2 [44] dataset and the resulting model is fine-tuned on the two considered emotion recognition datasets. For both the (S1) and (S2) models, ghost clusters are not considered within the aggregation phase (which is equivalent to applying a NetVLAD aggregation procedure).
- (S3) An emotion-recognition method that extends the SE-ResNet architecture with the GhostVLAD feature aggregation layer. The same multi-stage training used in Strategy 2 is employed here.
- (S4) A speech emotion identification framework that involves the SE-ResNet with the GhostVLAD layer and the classical triplet loss function with the SVM-RBF classifier on top.
- (S5) A proposed framework that involves multi-stage SE-ResNet training, GhostVLAD feature aggregation layer, and emotional metric learning (*cf.* Section 3.3) with the SVM-RBF classifier on top.

For the S1, S2 and S3 approaches, the SE-ResNet-34 architecture ends with two fully connected layers, one with 512 neurons, and the output layer with a number of neurons equal to the number of emotion classes. In all of these cases, the cross-entropy loss function (softmax loss) is used to train the network. 

The obtained experimental results are summarized in Table 2. The scores reported here are the average, minimum, maximum and standard deviation of the accuracy rate obtained over the 10 considered folds. 

The analysis of the results presented in Table 2 makes it possible to draw the following conclusions:

- The lowest accuracy scores (67% and 53%, respectively, for RAVDESS and CREMA-D datasets) are obtained by the baseline emotion recognition method, which trains the considered SE-ResNet architecture from scratch. The results can be explained by the relatively small size of the datasets, which is not sufficient for taking into account the complexity of the related audio emotional features.
- Multi-stage SE-ResNet training partially solves the above-mentioned problem. The use of additional speech data (VoxCeleb2) allows for obtaining models with a higher capacity. Thus, the multi-stage training increases the overall system performance with more than 6%.
- The extension of the SE-ResNet with a GhostVLAD layer produces more effective feature representations. From the experimental results, presented in Table 1, we observe that the introduction of the aggregation layer on top of the CNN architecture increases emotion recognition accuracy for both considered databases (4.97% and 1.78% for RAVDES and CREMA-D, respectively).
- Compared to softmax loss, the introduction of the triplet loss function offers an average improvement of 1.2%. As we employ semi-hard sampling where all the positive/negative spectrogram images are selected within a mini batch, the system converges faster and yields better results in terms of accuracy on both datasets.
- The best results (with accuracy scores of 83% and 64% on the RAVDESS and CREMA-D datasets, respectively) are obtained by the complete method that integrates the whole chain, with SE-ResNet, GhostVLAD aggregation layer and the emotional metric loss. The use of the emotion constraint significantly increases the system’s performance by reinforcing emotional clustering. Remarkably, the proposed framework outperforms the recognition rate provided by human observers with more than 24%.

**Figure 8:**
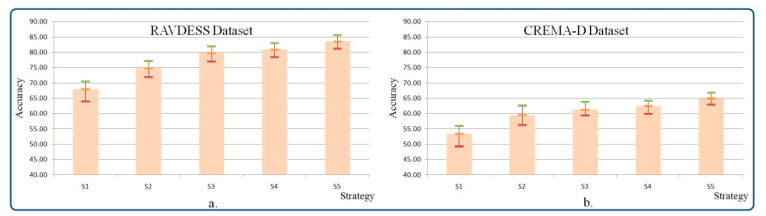
The statistical experimental results of the considered databases: (**a**) RAVDESS, (**b**) CREMA-D.

Figure 8 presents the accuracy variation, in terms of min, max and average scores, when considering 10-fold cross-validation on the (S1–S5) testing strategies. As can be observed, the experimental results are statistically consistent.

**Figure 9:**
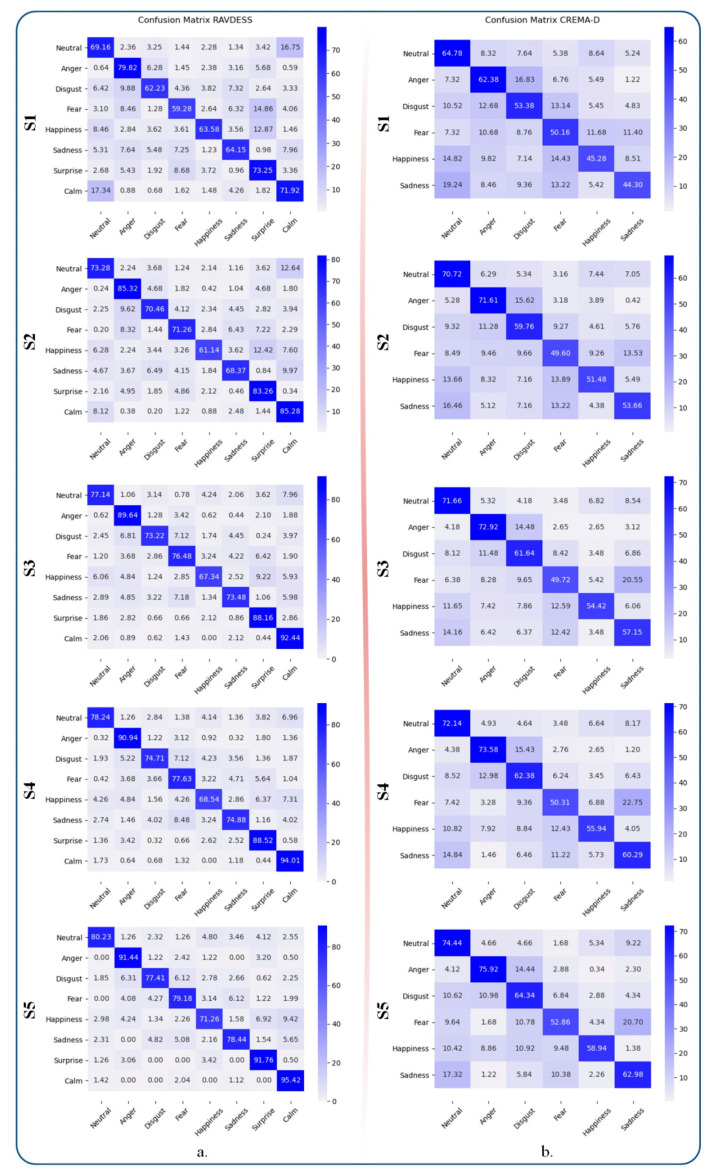
The confusion matrixes on the evaluation dataset (**a**) RAVDESS and (**b**) CREMA-D. (S1). The baseline method; (S2). SE-ResNet with multi-stage training; (S3). SE-ResNet with GhostVLAD layer; (S4). The SE-ResNet with the GhostVLAD layer and the classical triplet loss function; (S5). The proposed framework, which involves SE-ResNet with the GhostVLAD aggregation layer and emotion constraint loss.

Figure 9 presents the confusion matrices obtained on the two considered datasets. For RAVDESS, the highest performance score was obtained by the calm category, while the lowest accuracy rates were returned by the happy class for all of the considered test scenarios. For CREMA-D, the highest accuracy scores were obtained for the angry category and the lowest for the fear emotion in all scenarios.

When analyzing the experimental results obtained using RAVDESS (Figure 9a), it can be observed that the proposed framework (S5) recognizes anger, surprise and calm emotions with more than 90% accuracy, while the lowest accuracy score was obtained for happiness (around 70%). In addition, if we analyze the confusion matrices for the various classes involved, it can be observed that, for scenarios (S1–S4), the fear is confused with the surprise emotion and vice versa. 

However, this behavior is alleviated in (S5) by the emotional metric loss that takes into account the polarity between various classes of emotions. From Figure 9b, it can be concluded that, with CREMA-D, the proposed framework performs recognition well on the neutral and anger emotions. Particularly, its performance reaches 75.92% on the angry category. On the other hand, the system returns the lowest recognition scores on fear and happiness emotions with accuracy rates of less than 60%. This behavior can be explained by the enhanced relevance of the audio stream in predicting anger. Here, again, happiness cannot be reliably recognized. In our opinion, this shows the limitations of solely considering audio features, which can lead to ambiguities, notably in the case of categories with similar valences. One solution would be the integration of multiple modalities, i.e., audio and visual features, which can provide complementary information into the classification process.

**Figure 10:**
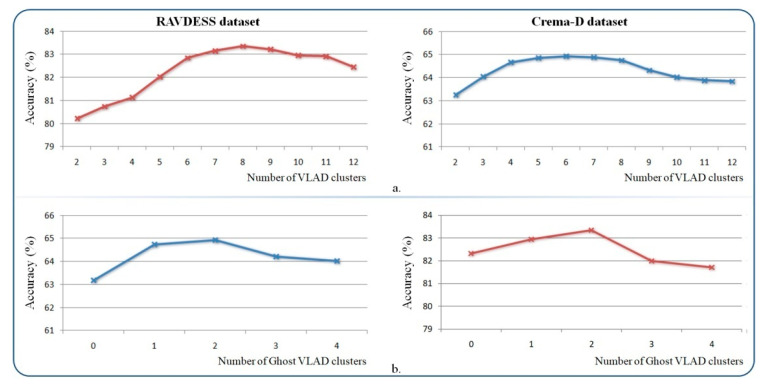
The system performance evaluation on RAVDESS and CREMA-D datasets with the different parameters involved: (**a**) the number of NetVLAD clusters (*K*) and (**b**) the number of GhostVLAD clusters (*G*).

We also evaluated the impact of the most important parameters involved in the system’s performance: the number of pertinent (*K*) and ghost clusters (*G*) within the GhostVLAD aggregation layer and the α, β control-margin parameters between various positive, related and negative samples within the emotion metric loss (Figure 10).

For the GhostVLAD layer, we found that K=8 and G=2 clusters led to the best results in the considered range. Too large or too low numbers of clusters would negatively affect the performances. Intuitively, the value of *K* should be compliant with the number of emotion categories. This implicitly indicates that the retained clusters correspond to the aggregation of the spectrograms to the emotional categories. The introduction of two ghost clusters brings about a significant improvement over NetVLAD. The ghost clusters enable the system to automatically down-weight the contribution of noisy speech segments. Using a low number of ghost clusters (one or two) improves recognition accuracy. Furthermore, increasing the number of ghost clusters (starting from 3–4) penalizes the overall performances, since, in this case, significant samples can be assigned to such ghost clusters.

**Figure 11:**
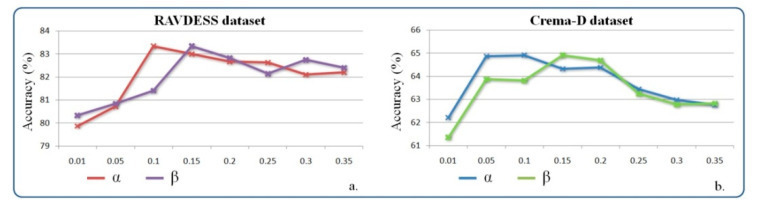
The system performance evaluation for different values of the control margins α and β on (**a**) RAVDESS dataset; (**b**) CREMA-D dataset.

Next, we investigated the influence of α and β (Equation (7)) margin parameters, which are involved in emotional metric loss. The obtained results are presented in Figure 11. Based on these results, we selected a value of 0.1 for α, and a value of 0.15 for β, which led to the best performances on both the RAVDESS and CREMA-D datasets. 

Finally, we compared the proposed emotion recognition framework with several state-of-the art techniques (Table 3). We retained, for comparison, a variety of emotional classification methods [8,25,26,32] that are based solely on audio features. For the method of Ghaleb et al. [23], which proposes a multi-modal framework, we only considered the results for the audio module. When available, we used the author’s code to evaluate the method. The parameters for all methods were selected to maximize the performance on the testing video dataset. Otherwise, we considered the results reported by the authors.

As can be observed, our approach yields a gain in accuracy within the range 3.49%–7.66% over the state-of-the-art techniques and on both benchmark datasets. Such high performances can be explained by the complexity and robustness of various modules involved in our framework, as well as by their combination within a unified workflow.

## 6. Conclusions

In this paper, we introduced a novel speech emotion-recognition approach. The proposed method is based on the Squeeze and Excitation ResNet (SE-ResNet) architecture, which is modified in a fully convolutional way in order to encode 2D spectrograms. The CNN architecture is extended with a GhostVLAD layer, used for feature aggregation to produce a compact, high-level feature representation of the audio stream. From a methodological point of view, the core of the approach relies on an extended triplet loss function with an *emotion constraint*, which is able to improve latent space data representation. Finally, the emotion class is predicted using an SVM classifier with RBF kernel.

The experimental evaluation carried out on the two publicly available datasets, RAVDESS [41] and CREMA-D [42], validates the proposed methodology, which yields recognition rates of more than 83% and 64%, respectively. When compared with the recognition rates obtained by human observers, the proposed methodology yields an increase in the global accuracy scores of more than 24%. Finally, the comparison with various state-of-the-art methods [8,23,25,26,32] demonstrates the superiority of the proposed methodology, with yields an increase in accuracy which ranges from 3.49% to 7.66%. 

In future work, we envisage further integrating a visual recognition module into the architecture. Our objective is to increase recognition performances by taking advantage of complementary information provided by the different visual and audio modalities. 

## Figures and Tables

**Table 1:** The 10-fold cross-validation scheme applied on the considered datasets.

Figure 1.		1	2	3	4	5	6	7	8	9	10
**CREMA-D**	**Train**	1—18	3—18	5—22	7—24	9—24; 1—2	11—24; 1—4	13—24; 1—6	15—24; 1—8	17—24; 1—10	19—24; 1—12
	**Val.**	19—21	21—23	23—24; 1	1—3	3—5	5—7	7—9	9—11	11—13	13—15
	**Test**	22—24	24; 1—2	2—4	4—6	6—8	8—10	10—12	12—14	14—16	16—18
**RAVDESS**	**Train**	1—73	10—82	19—91	28—91; 1—9	37—91; 1—18	46—91; 1—27	55—91; 1—36	64—91; 1—45	73—91; 1—54	82—91; 1—63
	**Val.**	74—82	83—91	1—9	10—18	19—27	28—36	37—45	46—54	55—63	64—72
	**Test**	83—91	1—9	10—18	19—27	28—36	37—45	46—54	55—63	64—72	73—81

**Table 2:** The experimental results presenting the influence of each component over the emotion recognition framework.

Method	Accuracy							
	RAVDESS [9]				CREMA-D [10]			
	**AVG %**	MIN %	MAX %	STD %	**AVG %**	MIN %	MAX %	STD %
Human observers	Not reported				**40.90**	-	-	-
(S1). Baseline method	**67.94**	63.95	70.48	2.33	**53.38**	49.25	56.02	2.12
(S2). SE-ResNet with multi-stage training	**74.81**	71.93	77.16	1.61	**59.47**	56.24	62.56	1.89
(S3). SE-ResNet with GhostVLAD layer	**79.78**	76.96	81.98	1.68	**61.25**	59.38	63.79	1.31
(S4). SE-ResNet with GhostVLAD layer + triplet loss	**80.93**	78.42	82.94	1.41	**62.44**	59.86	64.14	1.25
(S5). Proposed framework: SE-ResNet + GhostVLAD layer + emotion constraint	**83.55**	81.22	85.65	1.33	**64.92**	62.85	66.84	1.16

**Table 3:** Comparative experimental results of the proposed emotion recognition framework with various state-of-the-art methods.

Method	Accuracy	
	RAVDESS [41]	CREMA-D [42]
Bhavan et al. [25] 2019	75.69%	-
Issa et al. [26] 2020	71.61%	-
Kumar et al. [32] 2021	79.67%	58.72%
Ghaleb et al. [23] 2020	-	59.01%
Huang et al. [8] 2020	-	61.53%
**Proposed framework**	**83.35%**	**64.92%**
